# Clinical characteristics and STK11 gene mutations in Chinese children with Peutz-Jeghers syndrome

**DOI:** 10.1186/s12876-015-0397-9

**Published:** 2015-11-25

**Authors:** Zhiheng Huang, Shijian Miao, Lin Wang, Ping Zhang, Bingbing Wu, Jie Wu, Ying Huang

**Affiliations:** Department of Gastroenterology, Children’s Hospital of Fudan University, No. 399 WanYuan Road, Shanghai, 201102 China; The Molecular Genetic Diagnosis Center, Shanghai Key Lab of Birth Defects, Translational Medicine Research Center of Children’s Development and Disease, Pediatrics Research Institute, Children’s Hospital of Fudan University, Shanghai, 201102 China

**Keywords:** Peutz-Jeghers syndrome, STK11 gene, Mutation, Mucocutaneous pigmentation, Chinese children

## Abstract

**Background:**

Peutz-Jeghers syndrome (PJS) is a rare autosomal dominant inherited disease characterized by gastrointestinal hamartomatous polyps and mucocutaneous melanin spots. Germline mutation of the serine/threonine kinase 11 (STK11) gene are responsible for PJS. In this study, we investigated the clinical characteristics and molecular basis of the disease in Chinese children with PJS.

**Methods:**

Thirteen children diagnosed with PJS in our hospital were enrolled in this study from 2011 to 2015, and their clinical data on polyp characteristics, intussusceptions events, family histories, etc. were described. Genomic DNA was extracted from whole-blood samples from each subject, and the entire coding sequence of the STK11 gene was amplified by polymerase chain reaction and analyzed by direct sequencing.

**Results:**

The median age at the onset of symptoms was 2 years and 4 months. To date, these children have undergone 40 endoscopy screenings, 17 laparotomies and 9 intussusceptions. Polyps were found in the stomach, duodenum, small bowel, colon and rectum, with large polyps found in 7 children. Mutations were found in eleven children, including seven novel mutations (c.481het_dupA, c.943_944het_delCCinsG, c.397het_delG, c.862 + 1G > G/A, c.348_349het_delGT, and c.803_804het_delGGinsC and c.121_139de l19insTT) and four previously reported mutations (c.658C > C/T, c.890G > G/A, c.1062 C > C/G, and c.290 + 1G > G/A). One PJS patient did not have any STK11 mutations.

**Conclusions:**

The polyps caused significant clinical consequences in children with PJS, and mutations of the STK11 gene are generally the cause of PJS in Chinese children. This study expands the spectrum of known STK11 gene mutations.

## Background

Peutz-Jeghers syndrome (PJS, OMIM 175200) is a rare inherited autosomal dominant disorder characterized by mucocutaneous pigmentation (MP), hamartomatous polyposis and an increasing risk of developing cancer. The main clinical symptoms of this syndrome include abdominal pain, rectal blood loss, anemia, small bowel obstruction, and intussusception, leading to a high endoscopic and surgical resection rates. The first case of PJS was recognized by Peutz in 1921 in a Dutch family, and Jeghers et al. described the characteristics of this disorder in another family in 1948 [1]. PJS polyps commonly present in adolescence and early adulthood. One-third of affected individuals experience symptoms during the first 10 years of life [2]. Patients with PJS are at an increased risk of developing gastrointestinal cancer and extraintestinal neoplasms involving organs such as the ovaries, testes, breasts, pancreas, lungs, and uterine cervix [3].

The incidence of this disease has been estimated to be approximately 1 in 8,300 to 1 in 200,000 births. A germline mutation of the STK11 gene (also named LKB1), which is located on chromosome 19p13.3, is responsible for PJS. The STK11 gene consists of a 433-amino acid coding sequence comprised of nine coding exons and one non-coding exon.

To date, few studies have reported the clinical characteristics and STK11 gene of Chinese children with PJS [4]. In this study, we focused on the clinical characteristics and the detection of pathogenic germline mutations in Chinese children with PJS and their available family members in order to expand the spectrum of PJS gene mutations.

## Methods

### Patient and sample collection

The study protocol was approved by the ethics committee of the Children’s Hospital of Fudan University in Shanghai, China. The consent was obtained from all the parents for this study. Furthermore, consent to publish personal information such as that contained in Table 2 was also obtained from all the participants’ parents. "Thirteen children were enrolled in this study, including eleven boys and two girls, from February 2011 to February 2015. The ages of the children when they were diagnosed with the disease ranged from 16 month to 15 years old, and the age of disease onset ranged from birth to 5 years old. Blood samples were collected from four Chinese PJS families and eight sporadic patients.

Clinical diagnosis criterion: Each clinical diagnosis was made according to the following clinical criteria, and a patient presenting any one of the following symptoms was considered affected [5]:

1. Two or more histologically confirmed Peutz-Jeghers polyps.
2. Any number of Peutz-Jeghers polyps detected in one individual with a family history of PJS in a close relative(s).
3. Characteristic MP in an individual with a family history of PJS in a close relative(s).
4. Any number of Peutz-Jeghers polyps in an individual who also has characteristic MP.

**Fig. 1:**
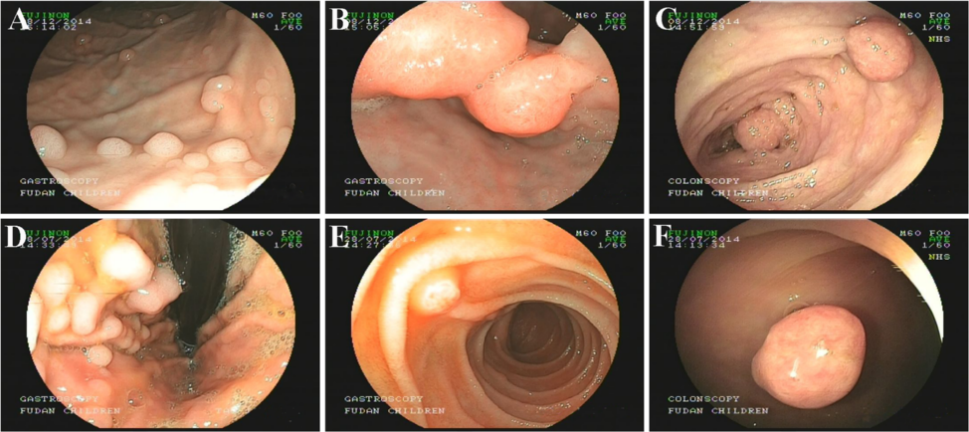
Endoscopy images of polyps in PJS patients. **a**-**c** Case 5 had polyps in the gastric body, duodenal bulb and transverse colon; **d**-**f** Case 8 had polyps in the gastric fundus, duodenal descending part and sigmoid colon

Endoscopy was performed to survey the children with PJS who had multiple polyps in the gastrointestinal tracts (Fig. 1). Various authors have proposed defining “large” polyps as 1 cm and “giant” polyps as those measure 2-cm and 3-cm in size [6–9]. In our study, polyps were classified as “large” if >1 cm in size and “giant” if >2 cm in size. The polyps were removed by polypectomy under endoscopy or laparotomy.

### Genomic DNA isolation and polymerase chain reaction (PCR)

Genomic DNA was extracted routinely from venous blood using an Isolation Kit (Qiagen, Germany) according to the manufacturer’s instructions. All nine coding exons and the flanking introns of the STK11 gene were amplified using the primers listed in Table 1. PCR of STK11 exons was performed in a 20-μl reaction volume that contained 10 μl of 2× PCR buffer, 3.2 μl of 0.25 mmol/l dNTPs, 0.5 μl of 10 μmol/l of each primer, 40 ng of genomic DNA, and 2 U Taq DNA polymerase (Takara LA Taq). PCR was performed under the following conditions: denaturation at 94 °C for 4 min, followed by 40 cycles at 94 °C for 30 s, 68 °C for 30 s, and 72 °C for 45 s.

**Table 1:** Primers for exon-specific sequencing of the STK11 gene

Exon	Forward	Reverse	Base
Exon 1	tccttttggggtttttgttg	ctggcacggaggacacag	576
Exon 2	tcccacagcactgtgaactc	attgccacaatggctgactt	394
Exon 3	ttcagaggggtggctgag	cagaagaatggcgtgaacct	488
Exon 4	gtgtgcctggacttctgtga	ccaccatctgccgtatgag	649
Exon 5	gtgtgcctggacttctgtga	ccaccatctgccgtatgag	649
Exon 6	ggtgtccttgagtccacagg	cagtcctctcaatgcctgct	384
Exon 7	ggagtggagtggcctctgt	acaggacactgcccagaga	400
Exon 8	atggctgagcttctgtggtc	ctttggggacgtgggatt	471
Exon 9	ggatacacctgggcctgac	caaaggccacatggcaac	485

### DNA sequencing

The PCR products were gel- and column-purified and directly sequenced. The purified PCR fragments were then sequenced using BigDye Terminator (Applied Biosystems, Foster City, CA, USA) on an ABI Prism 3500 genetic analyzer (Applied Biosystems).

### Bioinformatics analysis

Mutation Taster (http://www.mutationtaster.org/) was used to evaluate the disease-causing potential of the sequence alterations. We described the nucleotide and amino acid position in the STK11 gene according to the NCBI reference sequence (NG_007460.1).

## Results

### Clinical characters of children with PJS

These PJS children are from thirteen unrelated Chinese families from different area of China. Case 1 was from Anhui Province; Cases 3, 6 and 9 are from Jiangxi Province; Case 4 was from Zhejiang Province; Case 8 was from Shanghai; and Cases 10, 11 and 13 were from Jiangsu Province. All of these places are in eastern China. Case 2 was from Hunan Province, which is in south central China. Case 5 was from Sichuan Province, and Case 12 was from Hainan Province; both of these provinces in southwest China. Case 7 was from Gansu Province, which is in northwest of China.

The present age of children ranged from 2 to 17 years, and group included eleven boys and two girls. The clinical data, reasons for initial evaluation, clinical complication, etc. are shown in Table 2. The reasons for the children’s initial evaluations in our hospital are as follows, bloody stool (2/13), prolapsed polyps (1/13), intussusceptions (5/13), family history of PJS (4/13), MP (12/13) and anemia (3/13).

**Table 2:** Data on clinical character and complications of PJS children

Case no (sex)	Present age (y)	FH of PJS	Initial evaluation		Finding at first endoscopy/ surgery				Polyposis clinical complications					
			Age (y)	Reason	Age (y)	Method	Location	Size-no	Intussusception	Surgery	Anemia	Bloody stool	Abdominal pain	PRP
1 (M)	9	No	7	MP	7	EGD, Col	GD, Colon	Large-2	-	yes	-	-	-	-
2 (M)	9	No	5	MP, anemia	5	EGD	GD	Large-1	-	yes	62.2 g/L	-	-	-
3 (F)	7	No	3	MP, abdominal pain	3	Laparotomy	SB	Large-1	yes	yes	-	-	yes	-
4 (M)	13	No	9	MP, bloody stool	9	EGD, Col	GD, Colon	NA	yes	yes	-	-	-	-
5 (M)	15	Yes	13	MP, bloody stool	13	EGD, Col	GD, Colon	Large-1	-	-	81 g/L	-	-	-
6 (M)	11	Yes	9	MP, abdominal pain	9	Col	Colon	NA	yes	yes	-	-	yes	-
7 (M)	7	No	7	MP, abdominal pain	7	Laparotomy	SB	NA	yes	yes	99 g/L	-	-	-
8 (M)	15	Yes	8	FH,MP	8	Col	Col	Small	-	-	-	yes	-	-
9 (M)	17	No	8	MP, abdominal pain	8	x-ray	GD, Colon	unknown	yes	yes	-	-	yes	-
10 (M)	2	No	1	PRP	1	Col	Colon	Large-1	-	-	-	-	-	yes
11 (M)	7	Yes	6	MP, bloody stool	6	Laparotomy	Colon	Large-1	-	yes	-	yes	-	-
12 (M)	12	No	9	MP, abdominal pain	9	Col	Colon	Large-4	-	yes	-	-	yes	-
13 (F)	12	No	10	MP, abdominal pain	10	CE	SB	Small	-	-	-	-	yes	-

*CE* capsule endoscopy, *EGD* esophagogastroduodenoscopy, *Col* colonoscopy, *F* female, *FH* family history, *M* male, *MP* mucocutaneous pigmentation, *NA* not available, *PRP* prolapsed rectal polyp, *SB* small bowel

The median age at the onset of symptom was 2 years and 4 months. The median age at first screening test was 6.5 years (range 1–13 years). To date, there have been 40 endoscopy screenings (9 outside our hospital), 4 capsule endoscopies (1 outside our hospital), 17 laparotomies (5 outside our hospital), 9 intussusceptions (4 outside our hospital), and 2 intestinal obstructions (1 outside our hospital) in these children. In the group of children, 9 children had underwent laparotomies, 2 had underwent two laparotomies and 3 had underwent three laparotomies.

### Large polyp location and size

The thirteen children presented with variety of gastrointestinal polyps. During follow –up visits, polyps were located in the gastric body, antrum, duodenal bulb, descending duodenum, small bowel, transverse colon, descending colon, sigmoid and rectum. Case 1 had two large polyps in the sigmoid (4 × 3 cm) and stomach (2.3 × 1 cm), which were removed by polypectomy under endoscopy; a giant polyp in the stomach (12 × 6 cm) was removed by laparotomy. Case 2 had one giant polyp in the stomach (7 × 8 cm), which was removed by laparotomy. Case 3 required surgical intervention due to intussusception, which revealed 3 large polys of approximately 5 cm in diameter. Case 5 had two large polyps in the duodenal bulb (1.5 × 2 cm and 1 × 1 cm). Case 6 had two giant polyps in the descending part of the duodenum (3 × 0.5 cm, 2.5 × 2 cm) that were treated by endoscopic resection. Case 10 had a giant lobulated polyp in the sigmoid (4 × 2 cm) that was also removed under endoscopy. Case 11 had a giant polyp in the left colic flexure (3.5 × 6.8 cm). Case 12 presented with many polyps in the sigmoid colon, descending colon, ascending colon, et al.; and a total number of 9 large polyps with diameters ranging from 1 to 4 cm were removed by laparotomy or endoscopic resection separately. Case 13 showed only many small polyps in the small bowel under capsule endoscopy screen.

### Mutation analysis

**Fig. 2:**
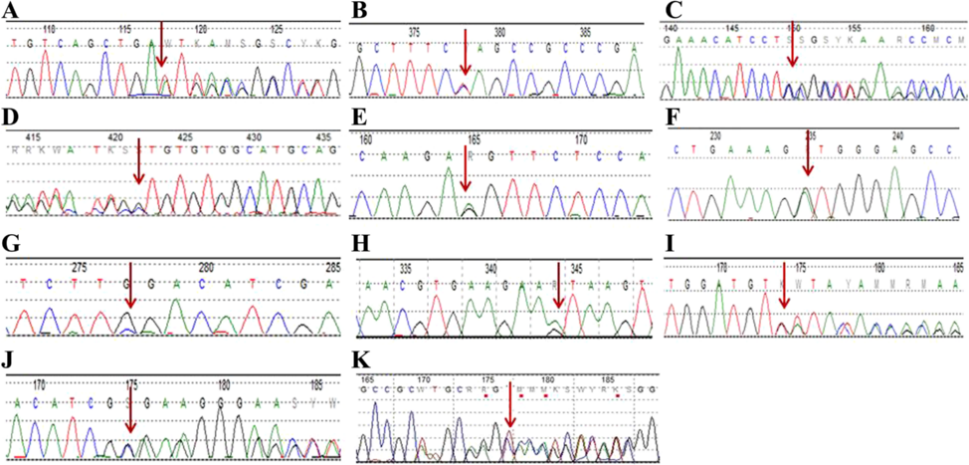
STK11 gene sequencing results. **a** The variant (481het_dupA) in exon 4 of the STK11 gene in Case 1. **b** Mutation c.658C > C/T in exon 5 of Case 2. **c** Mutation c.943_944het_delCCinsG in exon 8 of Case 3. **d** The variant (c.397het_delG) in exon 3 of the STK11 gene in Case 4. **e** The variant (c.890G > G/A) in exon 7 of the STK11 gene in Case 5. **f** The variant (c.862 + 1G > G/A) in intron 6 of the STK11 gene in Case 6. **g** Case 7 carries a c.1062C > C/G mutation in exon 8. **h** Mutation c.290 + 1G > G/A in intron 1 of Case 8. **i** The variant (c.348_349het_delGT) in exon 2 of Case 9. **j** Case 11 carries a c.803_804het_delGGinsC mutation in exon 6. **k** Case 12 carries a c.121_139del19insTT mutation in exon 1. The red arrows indicate the mutation sites

STK11 gene sequence analyses were performed for twelve of the thirteen pediatric PJS patients (Table 3). We identified eleven mutations, including seven novel mutations (four of which were de novo mutations) and four previously reported mutations (two of which were de novo mutations); one patient (Case 10) did not have any STK11 gene mutations. and Case 13 did not receive the genetic test. The seven novel mutations included six frameshift mutations (Case 1: 481het_dupA, Case 3: c.943_944 het_del CCinsG, Case 4: c.397het_delG, Case 9: c.348_349het_delGT, Case 11: c.803_804 het_del GG insC, and Case 12: c.121_139del19insTT) and one splice site mutation (Case 6: c.862 + 1G > G/A). The four previously reported mutations included one nonsense mutation (Case 2: c.658C > C/T), two missense mutations (Case 5: c.890G > G/A and Case 7: c.1062C > C/G), and one splice site mutation (Case 8: c.290 + 1 G > G/A) (Fig. 2).

**Table 3:** Summary of STK11 mutations identified in patients

Case no	Incidence	Age at onset	Variation type	Location	Variation position	Predicted consequence
1	Sporadic	1 y	Frameshift	Exon 4	c.481het_dupA	p.I161Nfs*2
2	Sporadic	2 y	Nonsense	Exon 5	c.658C>C/T	p.G220X
3	Sporadic	1 y	Frameshift	Exon 8	c.943_944het_delCCinsG	p.P315Gfs*21
4	Sporadic	7 y	Frameshift	Exon 3	c.397het_delG	p.V133Cfs*28
5	Family	3 y	Missense	Exon 7	c.890G>G/A	p.R297K
6	Family	2 y	Splice site	Intron 6	c.862+1G>G/A	-
7	Sporadic	6 y	Missense	Exon 8	c.1062C>C/G	p.F354L
8	Family	6 y	Splice site	Intron 1	c.290+1G>G/A	-
9	Sporadic	1 y	Frameshift	Exon 2	c.348_349het_delGT	p.L117Ifs*45
10	Sporadic	6 m	N	N	N	N
11	Family	6 y	Frameshift	Exon 6	c.803_804het_delGGinsC	p.G268Afs*19
12	Sporadic	2y	Frameshift	Exon 1	c.121_139del19insTT	p.K41Lfs*116
13	Sporadic	1y	ND	ND	ND	ND

Novel variants are in bold font

*N* negative, *ND* not detected

Regarding these novel mutations, Case 1 had an A base insertion at exon 4 (481het_dupA) that caused a frameshift mutation and resulted in a change in codon 161 from Ile to Asn and a termination at codon 163, leading to partial loss of the kinase domain and complete loss of the C-terminus. In Case 3, a 2-bp deletion was detected (c943_944) and one G base was inserted, which led to a Pro-to-Gly substitution at codon 315 and a partial loss of the C-terminus. Cases 12 and 13 had mutations similar to those of Case 3. In Case 4, a one-base deletion (c397het_delG) was detected in exon 3, which led to a Val-to-Cys substitution, with a premature stop at codon 161. Case 6 had a heterozygous de novo mutation with a G-to-A substitution at intron 6. Case 9 had a frameshift mutation with a 2-bp deletion (c.348–349delGT) in exon 2, which caused a Leu-to-Ile substitution at codon 117 and a premature terminator at codon 162, leading to partial loss of the kinase domain and a complete loss of the α-helix of the C-terminus. Case 2 possessed a novel mutation (c.658C > C/T) in exon 5 of the STK11 gene that resulted in the production of a truncated protein (Q220X). This mutation has never been reported in Chinese PJS patients.

**Fig. 3:**
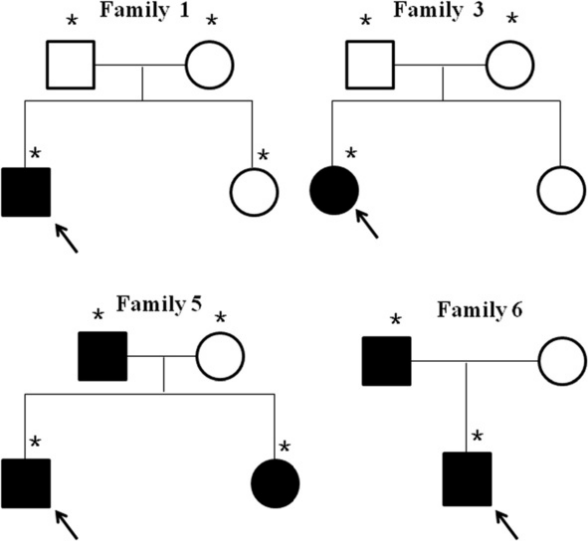
Pedigrees of the four PJS families (Case 1, 3, 5, 6). Square: male; circle: female; black symbol: affected individual; white symbol: unaffected individual. Asterisks indicate the family members with available genetic analyses. The proband in each pedigree is indicated by an arrow

Furthermore, the gene mutations and the histories of the available pedigree were also examined in the whole family of Cases 1, 3, 5 and 6 (Fig. 3). Case 5’s father and sister had the same mutation as Case 5. Case 6’s father had the same mutation as Case 6. There were no STK11 gene mutations detected in the father of Case 2. The father of Case 8 was also a PJS patient but could not be included because he died of colon cancer at age 39. The mother of Case 11 is also a PJS patient; however she refused the STK11 gene test.

## Discussion

PJS is a triad that includes MP, gastrointestinal polyposis and an increased risk of cancer. The polyposis often occurs in the intestinal tract but can appear anywhere. The complications of PJS include chronic abdominal pain, anemia due to polyp ulceration or infarction, bloody stools, and acute intestinal intussusceptions that require surgical intervention.

For children with PJS, the recent guidelines on when and how to screen for polyps vary in different countries. A Dutch surveillance recommendation suggests starting with capsule endoscopy at age 10 and continuing with capsule endoscopy every 2 to 3 years if no polyps are identified [10]. A French group recommended that regular surveillance is recommended every 2 years for PJS children over 8 years old with PJS [11]. An American group suggested that surveillance should begin with endoscopy at approximately 4 to 5 years of age [6]. Our research showed that the median age at first screening in Chinese children with PJS was 6.5 years. Because children have smaller lumen sizes than adults, smaller polyps may potentially cause an obstruction or an intussusception. Therefore, in our study, polyps were classified as “large” at >1 cm and “giant” at >2 cm. Our study showed that children with PJS had more endoscopic resections than laparotomies. These observations indicate that polypectomy with endoscopy is safe, efficacious, and cost-effective for the management of polyps in PJS patients. However, surgical resection may be employed for large polyps with non-polypoid morphology, polyps in anatomic locations that area not amenable to endoscopic removal, and intussusception, and intestinal obstructions that cannot be reversed [12].

Mutations in the STK11 gene, which is located on chromosome 19p13.3, are the causative agent in PJS patients. The STK11 protein is composed of three major domains: the N-terminal non-catalytic domain (encoded by amino acids 1–49), the catalytic kinase domain (encoded by amino acids 49–309) and the C-terminal non-catalytic regulatory domain (encoded by amino acids 309–433) [13]. The N-terminal domain contains the nuclear localization signal. The catalytic kinase domain forms a complex with STe20-Related Adaptor(STRAD) and scaffold protein 25 (MO25) to maintain the activation of this kinase [14]. Variations in PJS patients are mostly located in the catalytic domain region and cause dysfunctions in kinase activity, thus disrupting the function of STK11.

In our study, we identified STK11 mutations in sporadic cases and in family members of Chinese children with PJS. We reported seven novel mutations that have never been reported in any database or in any published articles. The different mutation types and sites in the STK11 gene correlate with different complication risks. Saloch et al. reported that truncation mutations in PJS are associated with a larger number of polyps, surgical interventions and cancers [15]. Our study similarly indicates that children with frameshift mutations had more gastrointestinal polyps and received more endoscopic screenings and more surgical interventions. Schumacher et al. [16] discovered that missense mutations in the C-terminus and in regions VIB-VIII of the protein were more frequently associated with malignancies. Wang et al. reported that mutations affecting protein kinase domain XI, encoded in part by exon 7, correlated with a 90 % (9/10) incidence of gastrointestinal polyp dysplasia [17]. Lim et al. suggested that mutations in exon 3 were associated with a higher risk of cancer [18]. In another study, Mehenni et al. found statistically significant evidence correlating mutations in exon 6 with a higher risk of cancer [19]. Thus, different exons and different mutation types may play different roles in the effects of PJS.

No mutations were detected in one child with PJS in our study. Germline mutations of STK11 can only be detected in only 50–70 % of PJS patients [20]. Partial or whole exon deletions can be identified through multiplex ligation-dependent probe amplification (MLPA) or through quantitative polymerase chain reaction (qPCR) in approximately 30 % of patients [21]. Other genes besides 19p13.3 may also carry mutations. For example, additional evidence has been found for the roles of the 19q13.4 locus or other variants in LKB1-negative PJS mutation carriers [22, 23]. Furthermore, high-throughput sequencing technology, such as whole-exon sequencing, may be a powerful tool for identifying these causative variants.

Of the four known STK11 mutations, the de novo p.G220X variant was found in Case 2, and it was located in the catalytic kinase domain of the STK11 protein. This mutations was previously reported in a study of Japanese adults [24], and. Loss of kinase activity is most likely responsible for the development of the PJS phenotype. Case 2 developed more gastric polyps and experienced anemia and underwent polypectomy three times using endoscopic hemoclips. In Case 5, the missense mutation c.890 > G/A changed Arg to Lys at codon 297. This de novo mutation of the STK11 gene was previously unreported in Chinese populations, although this mutation had been reported in a Dutch patient [25]. The missense mutation p.F354L in exon 8 of STK11 gene that was identified in Case 7 that had been previously reported only in Chinese adults [26] and the mutation site was located in the C-terminal non-catalytic regulatory domain. Case 7 has undergone polypectomy twice using endoscopic hemoclips, in addition to an operative intervention because of acute intussusception. Loss of the C-terminal domain of STK11 leads to the loss of cell polarity and hamartoma formation as a result of inappropriate overgrowth of differentiated cells. These mutations lead to impairments in both AMPK signaling and LKB1 polarity function [27]. Case 8 carried a de novo STK11 gene mutation that was previously unreported in Chinese populations, although it had been reported in a French population [28].

Genotype-phenotype correlation studies have been described in PJS patients. The frequency and spectrum of the cancer risk in those with PJS are more than 4 times the normal levels. The different germline variants of the STK11 gene predispose patients to different disease manifestations (e.g., numbers of polyps and times of onset of mucocutaneous pigmentation) and different risks of cancer. Some researchers have demonstrated that mutation types and locations are associated with the initial clinical presentations, surgical interventions and cancers experienced by PJS patients [15, 16]. However, those observations are based on relatively small patient numbers, and more data are needed for confirmation. Genetic diagnosis, in addition to surveillance by gastrointestinal endoscopy, is a useful tool in prenatal diagnosis, the clinical management of patients, and cancer surveillance. Long-term follow-up data are needed to establish the genotype/phenotype association with malignancy because these patients in present study are children, and cancer is more often observed in adults [15].

## Conclusion

Taken together, our data confirmed that polyps can occur at an early age and in all parts of the gastroenterological tract in children with PJS. We also found seven novel and four recurrent STK11 gene mutations in Chinese children with PJS. These results expand the data on Chinese children with PJS with a variety of STK11 variants.
